# Natural progression of tarsal osteochondrosis in Standardbred pacers and trotters

**DOI:** 10.1111/vsu.70073

**Published:** 2026-01-03

**Authors:** Annette M. McCoy, Christine T. Lopp‐Schurter, Rebecca C. Bishop, Amy Narotsky, Kyle Grogger, Ann M. Kemper

**Affiliations:** ^1^ Department of Veterinary Clinical Medicine University of Illinois Urbana‐Champaign Urbana Illinois USA; ^2^ Department of Veterinary Clinical Sciences Iowa State University Ames Iowa USA; ^3^ Hoosier Equine Veterinary Services Anderson Indiana USA; ^4^ Brazos Valley Equine Hospitals Navasota Texas USA

## Abstract

**Objective:**

To determine the natural progression of tarsal osteochondrosis (OC) in a cohort of Standardbred foals and assess the impact of gait preference (trotting vs. pacing).

**Study design:**

Longitudinal observational cohort study.

**Animals:**

Client‐owned Standardbred foals (*n* = 148).

**Methods:**

Tarsal radiographs were taken every 2 months from 2 to 12 months of age and foals were video monitored to document time spent pacing or trotting. Differences between groups were assessed using χ^2^ analysis. Survival analysis was used to determine if lesion healing differed between groups over time.

**Results:**

Of 148 horses, 103 (69.6%) had OC lesions at 2 months of age but only 32 (21.6%) still had OC lesions by 12 months. In 28 of these horses, the lesions had progressed to osteochondrosis dissecans (OCD). Of 71 horses with lesions that healed, 60 (84.5%) did so by 6 months of age. Gait was not associated with presence or absence of OC lesions or healing of lesions over time. Foals spent less than 1% of their observed time trotting or pacing.

**Conclusion:**

Tarsal OC lesions were prevalent in this cohort of young Standardbreds, and most lesions healed within the first few months of life. There was no evidence for direct biomechanical forces from movement at the pace or trot affecting the location of OCD fragment development.

**Clinical significance:**

The critical window for tarsal OC lesion healing was prior to 6 months of age and further investigation of risk factors present during this time frame is warranted.

**Abbreviations**
DIRT: distal intermediate ridge of the tibia
DLPMO: dorsolateral‐plantaromedial oblique
DMPLO: dorsomedial‐plantarolateral oblique
IACUC: institutional animal care and use committee
LTR: lateral trochlear ridge of the talus
MM: medial malleolus of the tibia
MTR: medial trochlear ridge of the talus
OC: osteochondrosis
OCD: osteochondrosis dissecans

## 1 INTRODUCTION

Osteochondrosis (OC) is a commonly diagnosed developmental orthopedic disease characterized by delayed endochondral ossification. In horses, predilection sites include the tarsus (distal tibia and talus), stifle (trochlear ridges), and metacarpo/metatarsophalangeal joints (dorsal midsagittal ridge).^1^ Both genetic and environmental factors contribute to the development of OC, and certain breeds, including Standardbreds, are considered predisposed.^2^, ^3^, ^4^ Radiographically evident OC lesions (osteochondrosis manifesta) may eventually ossify and be considered healed, whereas others progress to osteochondral fragment formation, termed osteochondrosis dissecans (OCD).^5^ The determinants of lesion healing versus progression to OCD are poorly understood, but biomechanical forces may play a role, as early life exercise has been associated with OC lesion distribution and severity.^6^ Differences in OC lesion prevalence and distribution between Standardbred pacers and trotters have been reported,^7^, ^8^ but whether these differences reflect genetic risk, gait‐related biomechanical forces, or a combination of both remains unclear.

The objective of this study was to document the natural progression of tarsal OC in a cohort of Standardbreds during the first year of life and to determine if gait preference affected OC lesion distribution or the progression of OC lesions to OCD. It was hypothesized that biomechanical differences in natural gait patterns between pacers and trotters would result in different distributions of lesions and alter their propensity to progress to OCD.

## 2 MATERIALS AND METHODS

*Study cohort and radiographs:* The study cohort included Standardbred foals born at three farms in central Illinois between 2015 and 2018. Foals were enrolled in the study at 2 months of age, and radiographs were taken every 2 months until 12 months of age. Radiographs were obtained on the farms under institutional animal care and use committee (IACUC) approval (protocols #15086 and 18055) and with informed owner consent. Routine physical restraint (halter, lead rope, with or without lip twitch) was provided by trained farm personnel. Sedation (xylazine 0.3 mg/kg IV ± butorphanol 0.01 mg/kg IV ± acepromazine 0.02 mg/kg IV) was administered as needed for safety at the discretion of the farm manager and attending veterinarian. Four standard radiographic projections were obtained for each tarsus: dorsal–plantar, lateral, dorsomedial–plantarolateral oblique (DMPLO), and dorsolateral–plantaromedial oblique (DLPMO). Digital images were stored in the hospital repository at the University of Illinois Veterinary Teaching Hospital, where they were accessible to study personnel via commercial visualization software (Carestream Solutions version 12.1.0.1059, Carestream Health, Rochester, New York).

Radiographs were reviewed by a single board‐certified large animal surgeon (AMM). Tarsal radiographs were evaluated for the presence or absence of OC lesions, defined as a flattening or divot in the normal contour of the distal intermediate ridge of the tibia (DIRT), medial malleolus of the tibia (MM), and/or lateral or medial trochlear ridge of the talus (LTR, MTR), with or without a discrete osteochondral fragment (OCD) present.

*Field monitoring:* Foals were video monitored in their normal home environment for at least two consecutive hours each week from 2 to 12 months of age. As foaling was spread out over several weeks, this resulted in 49 weeks of video monitoring per farm, each year. Observation times varied by time of day (7:00 a.m.–7:00 p.m.) and day of the week (Monday to Saturday) to provide a representative assessment of activity. Videos were reviewed by two trained observers (AN and KG), who assigned an activity category to each foal at 30 s intervals. These categories were: nursing, eating/grazing, walking, trotting, pacing, cantering/galloping, standing quietly, and lying down. Weekly exercise was calculated as the percentage of time spent moving (walking, trotting or pacing, or cantering or galloping) divided by total observation time.

*Statistical analysis:* Statistical analyses were performed in the R computing environment^9^ using the basic *stats* package as well as the *survival* ^10^, ^11^ and *survminer* ^12^ packages. Comparisons between groups were performed using χ^2^ analysis; for comparisons between two groups, this was a two‐sample test with continuity correction, while for comparisons among more than two groups it was a χ^2^ goodness‐of‐fit test with the null assumption of equal proportions between groups. Survival analysis was used in affected foals to determine if there was a difference in lesion healing between groups over time. For this analysis, “survival” was defined as lesion persistence. For comparison of activity between farms and time intervals, a two‐way ANOVA was performed with a post hoc Tukey HSD test for multiple comparisons of means. Significance for all tests was set at *p* < .05.

## 3 RESULTS

*Study cohort:* A total of 158 foals from the three farms were enrolled at 2 months of age; of these, 148 completed the study (Supporting Information, Table S1). Reasons for exiting the study included being sold or sent off the farm (*n* = 9) and death (*n* = 1). All foals had at least four complete sets of radiographs available for review (of six possible sets). One farm restricted access to the foals around weaning time, and radiographs at 4 months of age were therefore unavailable for this subgroup (*n* = 58). Foals not radiographed at 4 months were conservatively assumed to show no lesion healing between 2 and 4 months of age. Consistent with the general Standardbred population in central Illinois, pacers were overrepresented, with 101 pacers (68.2%) and 47 trotters (31.8%). There were 82 (55.4%) colts and 66 (44.6%) fillies in the cohort, with no difference in sex distribution between gaits (55/101 [54.5%] pacers were colts, 27/47 [57.4%] trotters were colts; *p* = 0.87).

*Prevalence of tarsal OC and progression over time:* At 2 months of age, 103/148 (69.6%) of the horses had one or more tarsal OC lesions. By 12 months of age, only 32/148 (21.6%) of the horses had lesions; 24/32 (75%) of the horses had DIRT lesions (22 OCD, 2 OC), 5/32 (15.6%) had LTR lesions (4 OCD, 1 cystlike lesion), 2/32 (6.3%) had both DIRT and LTR lesions (1 DIRT OC/LTR OCD, 1 DIRT OCD/LTR cystlike lesion), and 1/32 (3.1%) had a MM cystlike lesion. Twelve horses (37.5%) had bilateral lesions. Of the 32 affected horses at 12 months of age, 23 were pacers (18 DIRT, 4 LTR, 1 DIRT and LTR) and nine were trotters (6 DIRT, 1 LTR, 1 DIRT + LTR, 1 MM). There was no difference in the prevalence of OC lesions between pacers and trotters at any time point (*p* > .28; Table 1), and no difference in lesion distribution (*p* = .82). When considering OC lesion prevalence at 2 and 12 months, there was no difference between farms (*p* = .88 at 2 months, *p* = .17 at 12 months) or between sexes (*p* = .61 at 2 months, *p* = .27 at 12 months).

**TABLE 1:** Prevalence of osteochondrosis lesions in 148 Standardbred foals, by gait, from 2 to 12 months of age. Results are presented as number affected (percentage).

Age (months)	Pacers (*n* = 101)	Trotters (*n* = 47)	*p*
2	67 (66.3)	36 (76.6)	.28
4	25 (43.9) (*n* = 57)	18 (54.5) (*n* = 33)	.45
6	30 (29.7)	15 (31.9)	1
8	27 (26.7)	11 (23.4)	.82
10	25 (24.8)	9 (19.1)	1
12	23 (22.8)	9 (19.1)	.78

*Note*: One farm did not allow radiographic evaluation at 4 months of age due to weaning. The *p*‐value reflects difference in proportions as calculated by a two‐sample χ^2^ test with continuity correction.

**FIGURE 1:**
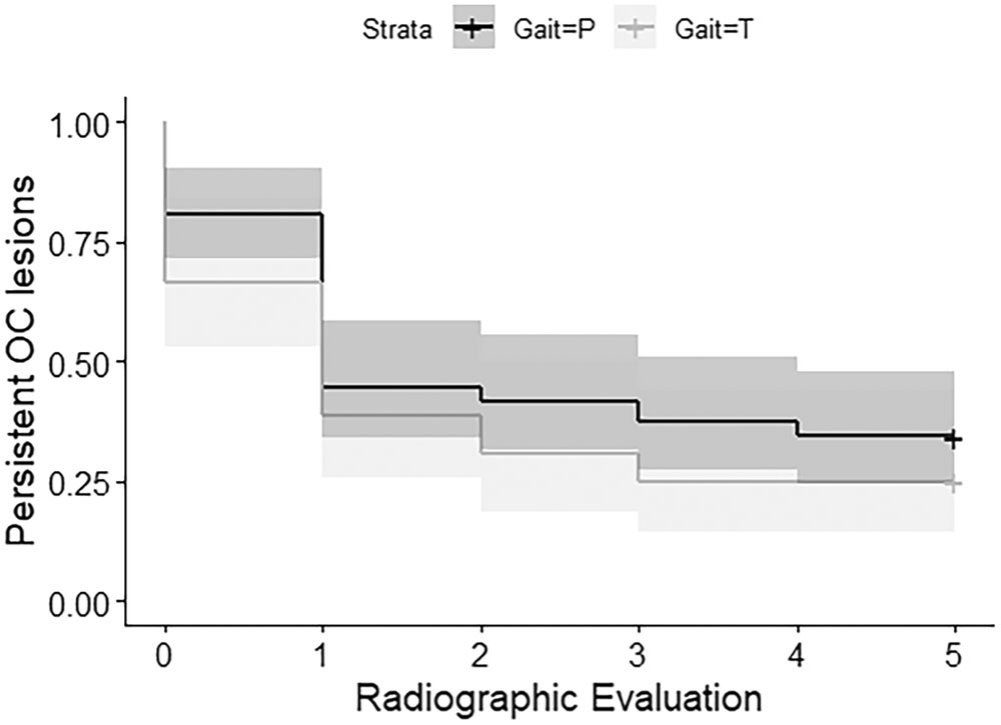
Kaplan–Meier curve showing healing of osteochondrosis (OC) lesions in 103 Standardbred pacers (black line) and trotters (gray line). Shaded areas surrounding each line show 80% confidence interval. Baseline (0) radiographics were obtained at 2 months of age; follow‐up radiographic evaluations (1–5) were performed every 2 months until 12 months. A subset of foals was not radiographed at 4 months of age; their lesions were conservatively assumed not to have healed between 2 and 4 months of age. No difference in healing was observed between groups over time.

**FIGURE 2:**
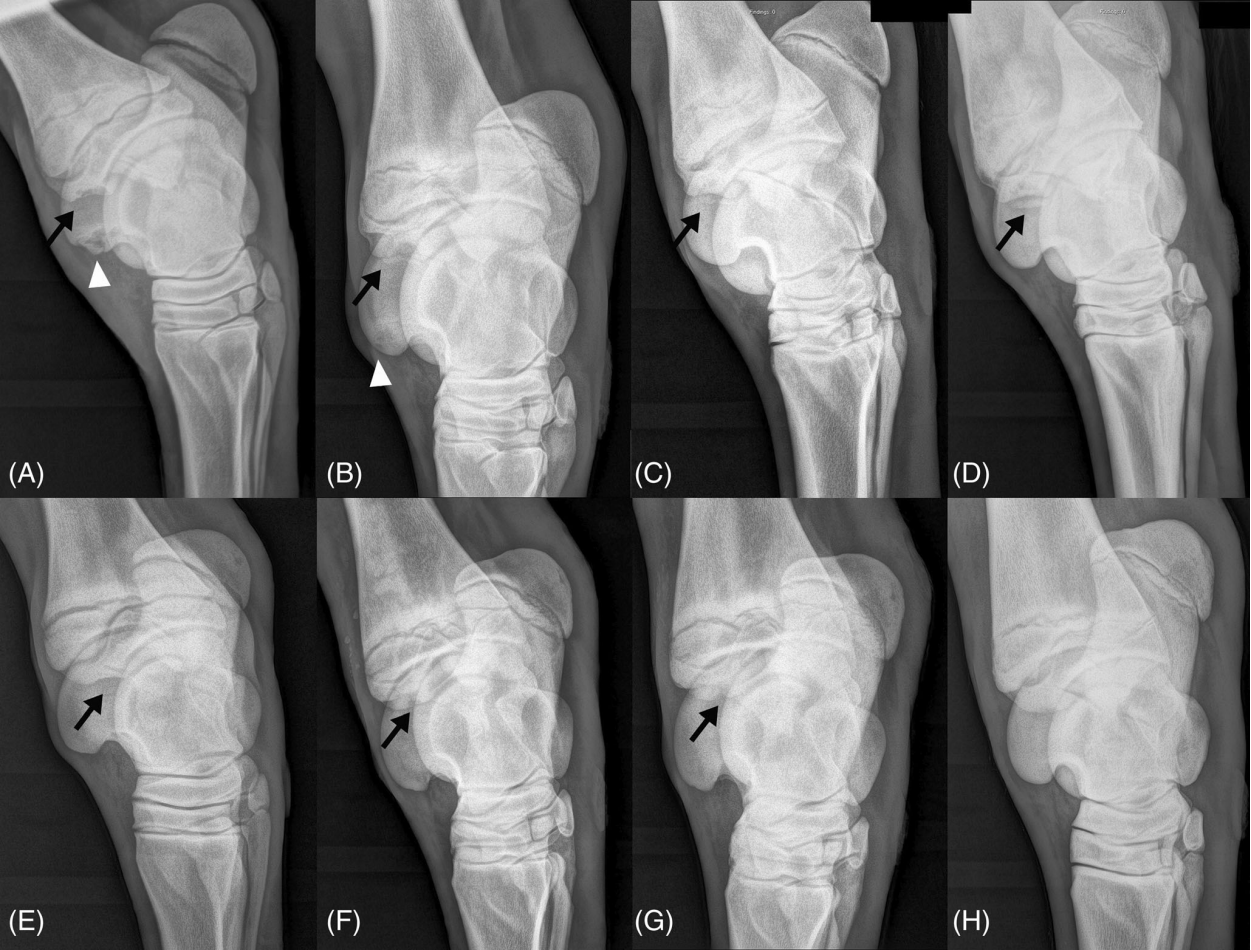
Radiographs showing tarsal lesion progression in two foals from 2 to 12 months of age. (A–D) Dorsomedial‐plantarolateral oblique (DMPLO) images of the left tarsus of a foal at 2, 4, 6, and 12 months of age showing progression of a distal intermediate ridge of the tibia (DIRT) lesion from osteochondrosis (OC) to osteochondrosis dissecans (OCD) (black arrows), and healing of a lateral trochlear ridge of the talus (LTR) lesion (white arrowheads). A DIRT osteochondral fragment was initially visible at 6 months of age and persisted at 12 months, whereas the LTR lesion had completely healed by 6 months of age. (E–H) Dorsomedial‐plantarolateral oblique images of a second foal at 2, 4, 6, and 12 months of age showing healing of a DIRT OC lesion. Although a small bone concavity was still present at 6 months of age, there was no fragmentation and the lesion had completely healed by 12 months.

Survival analysis revealed no difference in healing of lesions over time between pacers and trotters (*p* = .19; Figure 1, Table 2). There were also no differences in healing between groups when survival analysis was performed comparing sex (*p* = .19), farm (*p* = .06), and foaling year (*p* = .64) (Supporting Information, Tables S2–S4). When only affected horses were considered, pacers had a higher proportion of individuals with DIRT lesions at 2 months of age than trotters (56/67 vs. 17/36, *p* = .0003), but no such difference persisted at 12 months (18/23 vs. 7/9, *p* = 1). Among the 71 horses with lesions identified at 2 months that eventually healed, 60/71 (84.5%) healed by 6 months of age. Once an OCD fragment was present, no healing occurred. Osteochondrosis dissecans (OCD) fragments were noted as early as 4 months of age, and commonly by 6 months of age (Figure 2).

**TABLE 2:** Survival analysis statistics for healing of osteochondrosis lesions in Standardbred foals, by gait, from 2 to 12 months of age. For this analysis, “survival” was defined as lesion persistence.

Exam number	Age (months)	Horses at risk	Horses healed in interval	Proportion affected at end of interval	Standard error	95% CI
Pacers						
0	2	67	13	0.81	0.05	0.72, 0.91
1	4	54	24	0.45	0.06	0.34, 0.58
2	6	30	2	0.42	0.06	0.32, 0.55
3	8	28	3	0.37	0.06	0.27, 0.51
4	10	25	2	0.34	0.06	0.25, 0.48
5	12	23	0	0.34	0.05	0.25, 0.48
Trotters						
0	2	36	12	0.67	0.08	0.53, 0.84
1	4	24	10	0.39	0.08	0.26, 0.59
2	6	14	3	0.31	0.08	0.19, 0.50
3	8	11	2	0.25	0.07	0.14, 0.44
4	10	9	0	0.25	0.07	0.14, 0.44
5	12	9	0	0.25	0.07	0.14, 0.44

*Note*: Only foals with osteochondrosis (OC) lesions diagnosed radiographically at 2 months of age (*n* = 103) are included in this analysis. Exam 0 is at the time of enrollment. Foals that were not radiographed at 4 months of age were conservatively assumed not to have healed their lesions between 2 and 4 months of age. The *p* value for comparison between groups was .19.

**FIGURE 3:**
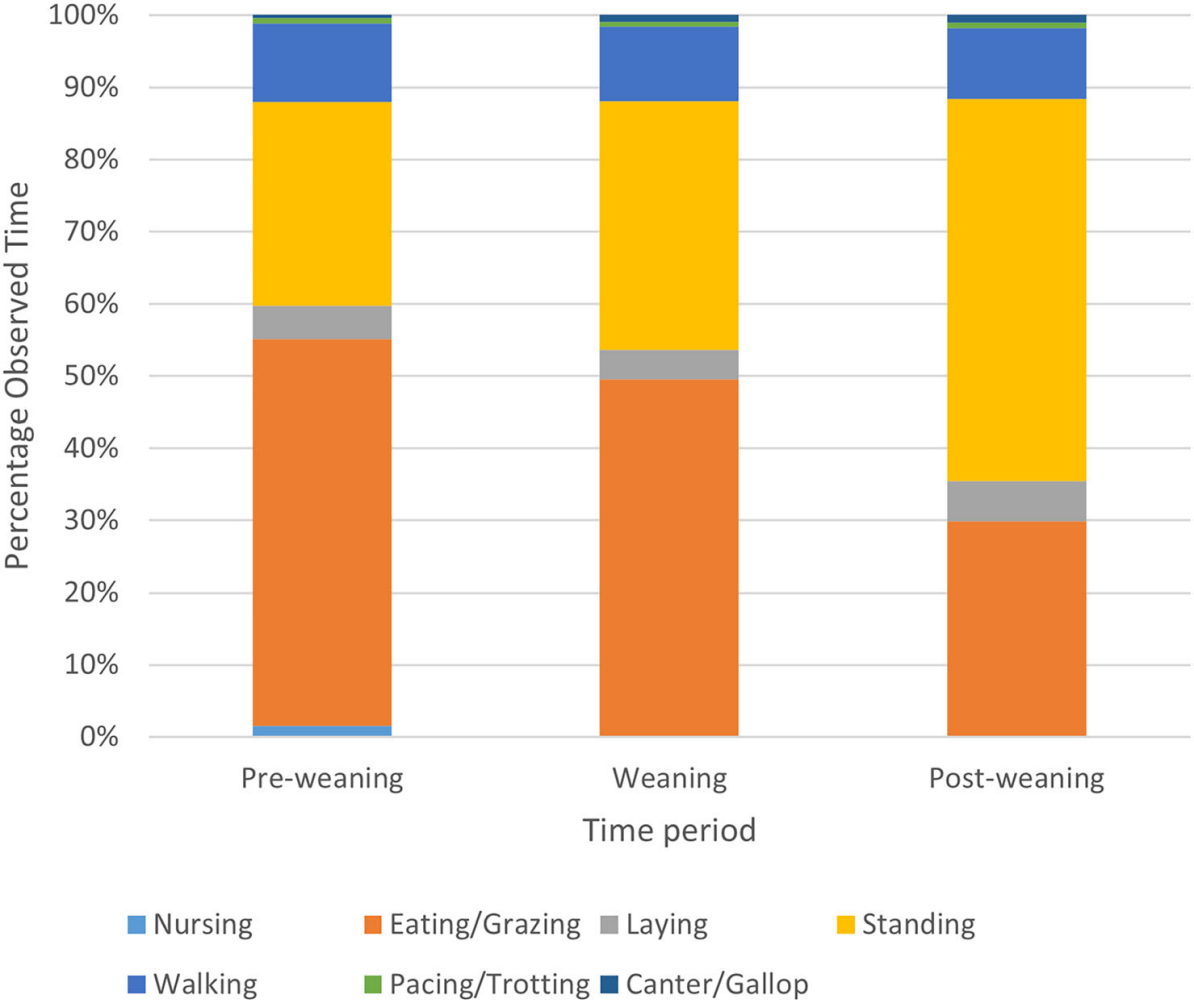
Activity during the preweaning (observational weeks 1–15), weaning (observational weeks 16–22), and postweaning (observational weeks 23–49) periods, averaged across all foals, shown as a percentage of observed time spent in each activity category. Foals spent most of their time eating or standing quietly in all three time periods.

*Observed activity:* Supporting Information, Table S5;shows a summary of activity by week. Prior to weaning, foals at all three farms were maintained in large pastures with their mares. At the time of weaning, management practices diverged. One farm kept weanlings in sex‐segregated pastures, where they remained until sold as yearlings. The second moved weanlings into sex‐segregated large dry lots for several months and then back into sex‐segregated pastures. The third farm kept weanlings stalled for several weeks at the time of weaning and then moved them to a facility where the sex‐segregated dry lots were adjacent to small pastures into which daily turnout was provided. To facilitate assessment across the farms and years, activity was thus divided into the preweaning period (observational weeks 1–15), the weaning period (observational weeks 16–22), and the postweaning period (observational weeks 23–49) (Figure 3; Supporting Information, Table S6). Across all three periods, foals spent most of their time eating and standing quietly. These two activities combined accounted for approximately 80% of the observed time. Most activity was walking, accounting for 10% of the observed time. Foals spent 2% or less of their observed time trotting/pacing or cantering/galloping, and these activities were exhibited in short bursts rather than as sustained behavior. When considered over the entire study period, there were no differences between farms for any of the observed activities (*p* > .07). However, as noted above, one farm stalled its foals during weaning period; thus, although it has no data for this time period included in the statistical analysis, its foals were clearly limited in their activity in a way that the foals at the other farms were not. As expected, nursing behavior was almost exclusively observed in the preweaning period (*p* = .001). Foals spent more time standing quietly in the postweaning period than in either of the other two time periods (*p* = .001) and there was a corresponding decrease in grazing behavior (*p* = .0007).

## 4 DISCUSSION

The overall incidence of tarsal OC lesions in this study cohort was similar to previous reports in Standardbreds,^7^, ^13^ but contrary to the study's hypothesis and previous reports^7^, ^8^ no difference was found in lesion distribution between pacers and trotters at 12 months of age. Based on the very limited time spent moving at the pace or trot, it seems unlikely that direct biomechanical effects of either gait played a role in the healing (or otherwise) of OC lesions in this cohort, but this cannot be completely ruled out. It is possible that the cumulative effect of time spent at one gait or the other over the first few months of life is more important than any single exercise event. It is noteworthy that the progression of tarsal OC lesions in this cohort closely mirrors that previously reported in a small cohort of Warmblood foals,^14^ with most lesions that ultimately healed having done so by 6 months of age. This supports the concept of a critical time window for OC lesion healing across horse breeds, regardless of the typical time frame in which lesions are diagnosed. Continued investigation into risk factors that may be present during this time frame is warranted.

The major clinical relevance of these findings relates to recommendations for surgical intervention for OC. Once fragmentation (OCD) developed in a joint in the study cohort, further healing did not occur. In such cases early surgical removal (i.e., before12 months of age) could be a reasonable option if substantial effusion or lameness is also present. In contrast, foals with abnormal bone contours but no fragmentation often continued to show radiographic improvement through 12 months of age. In these cases, delayed surgical intervention (i.e., after 12 months of age) would be reasonable, with periodic radiographic screening until full healing is achieved or an osteochondral fragment develops. This conclusion is supported by a recent study in young Walloon sport horses in which 1/6 DIRT lesions detected at 1 year of age progressed from OC to OCD by 2 years of age. The five DIRT lesions in this cohort that already had fragmentation present at a year of age remained static at 2 years of age.^15^

Osteochondrosis is known to be a complex disease, with the effects of genetics and environment both playing a role in the development of lesions.^16^ Previous work evaluating the influence of exercise on OC development has been somewhat equivocal and has been focused on the first few months of life. In a landmark study of 43 Warmblood foals bred to be genetically predisposed to OC, the number of OC lesions did not differ between foals that were box‐rested, those that were box‐rested with training, and those that were pasture raised during the first 5 months of life.^6^ However, there was a tendency towards more severe OC lesions in the box‐rested group at 5 months of age, and the authors concluded that pasture raised conditions were preferred throughout the first year of life. In another large cohort study of 378 foals of several breeds, irregular or limited exercise was associated with greater severity of OC lesions compared to foals with daily turnout.^2^ There was a clinical impression during the course of the current study that there was a difference in prevalence of persistent OC lesions between the three farms that contributed to the study cohort, although the difference did not reach statistical significance. All of the farms managed their foals on pasture until the time of weaning, around 5 months of age, at which point their management practices diverged. The farm that stalled its foals during weaning had no foals with lesions that improved radiographically after 6 months of age, whereas both of the other farms, which kept foals on daily turnout in dry lots or pasture, had several foals that demonstrated radiographic healing between 6 and 12 months of age. The role of exercise during and after weaning on the healing or progression of OC lesions bears further examination in a larger cohort.

This study had several limitations. Trotters were underrepresented in this cohort, and the number of affected individuals at 12 months of age was small, which limited the experiment's capacity to distinguish outcomes between gaits. The small number of affected individuals also likely limited the capacity of the experiment to detect impacts of the marked management differences between farms on outcomes. Field observations of the study cohort were limited to 2 h a week for practical reasons related to the need for personnel to travel to the farms, and so periods of greater activity might have been missed. Although the time of day and day of the week for weekly observations were deliberately varied throughout the study to help minimize this limitation of study design, it is possible that the findings might have been different if gait observations were more extensive. Ideally, future studies would fit the foals with sensors to record data continuously. All the foals in the current study, raised within a 100 mile radius, were fed locally sourced hay and were of similar genetic backgrounds but there were undoubtedly unmeasured genetic and environmental factors that played a role in the manifestation and progression of disease.

In conclusion, tarsal OC lesions were highly prevalent in this cohort of young Standardbreds, and most lesions healed within the first few months of life. Serial radiographic examinations should be used to monitor healing or progression of disease through at least 12 months of age, and the presence of an OCD fragment should be a key consideration when making decisions about early surgical intervention (i.e., before 12 months of age). There was no evidence for direct biomechanical forces from movement at the pace or trot affecting OCD fragment development, although investigation in a larger cohort with continuous gait monitoring would be needed to confirm this finding. The critical window for lesion healing seems to be prior to 6 months of age and further investigation of risk factors present during this time frame, including access to daily turnout during the weaning period, is warranted.

## AUTHOR CONTRIBUTIONS

McCoy AM, DVM, MS, PhD, DACVS (Large Animal): Contributed to conception and design, data acquisition, analysis and interpretation, manuscript preparation, and approved the final version of the manuscript. Lopp‐Schurter CT, DVM, DACVS (Large Animal): Contributed to data acquisition and approved the final version of the manuscript. Bishop RC, DVM, MS, PhD, DACVS (Large Animal): Contributed to data acquisition, analysis, and interpretation and approved the final version of the manuscript. Narotsky A, DVM: Contributed to data acquisition, analysis, and interpretation, and approved the final version of the manuscript. Grogger K, DVM: Contributed to data acquisition, analysis, and interpretation, and approved the final version of the manuscript. Kemper AM, DVM: Contributed to data acquisition and approved the final version of the manuscript.

## FUNDING INFORMATION

This work was supported in part by Morris Animal Foundation (D16EQ‐311, D20EQ‐046) and United States Department of Agriculture (USDA) Hatch Funds (ILLU‐888‐349). Student salary support was provided by the Office of the Director, National Institutes of Health (T35 OD011145).

## CONFLICT OF INTEREST

The authors declare no conflict of interest related to this report.

## Supporting information


**Supplementary TABLE S1:** Distribution of foals between farms across the years of study enrollment.
**Supplemental TABLE S** **2**: Survival analysis statistics for incidence and healing of osteochondrosis lesions in 103 Standardbred foals, by sex, from 2 to 12 months of age.
**Supplemental TABLE S** **3**: Survival analysis statistics for incidence and healing of osteochondrosis lesions in 103 Standardbred foals, by farm, from 2 to 12 months of age.
**Supplemental TABLE S** **4**: Survival analysis statistics for incidence and healing of osteochondrosis lesions in 103 Standardbred foals, by foaling year, from 2 to 12 months of age.
**Supplemental TABLE S** **5**: Summary of observed activity by observation week. Percentages shown for each activity are the concatenation of all horses, farms, and years and are presented as median [interquartile range]. Foals were enrolled in the study at 8 weeks of age, and within each week's observations (after Week 1) there was a range in the ages of the observed foals because foaling at each farm was spread out over several weeks.
**Supplemental TABLE S** **6**: Summary of activity in percentage of observed time by farm and time period (pre‐weaning, weaning, post‐weaning).

## References

[1] Kane AJ , Park RD , McIlwraith CW , Rantanen NW , Morehead JP , Bramlage LR . Radiographic changes in thoroughbred yearlings. Part 1: prevalence at the time of the yearling sales. Equine Vet J. 2003;35:354‐365. 12880003 10.2746/042516403776014280

[2] Lepeule J , Bareille N , Robert C , et al. Association of growth, feeding practices and exercise conditions with the prevalence of developmental Orthopaedic disease in limbs of French foals at weaning. Prev Vet Med. 2009;89:167‐177. 19329202 10.1016/j.prevetmed.2009.02.018

[3] Philipsson J , Andreasson E , Sandgren B , Dalin G , Carlsten J . Osteochondrosis in the tarsocrural joint and osteochondral fragments in the fetlock joints in Standardbred trotters. II. Heritability. Equine Vet J Suppl. 1993;16:38‐41.

[4] McCoy AM , Norton EM , Kemper AM , Beeson SK , Mickelson JR , McCue ME . SNP‐based heritability and genetic architecture of tarsal osteochondrosis in north American Standardbred horses. Anim Genet. 2019;50:78‐81. 30353927 10.1111/age.12738

[5] McCoy AM , Toth F , Dolvik NI , et al. Articular osteochondrosis: a comparison of naturally‐occurring human and animal disease. Osteoarthr Cartil. 2013;21:1638‐1647. 10.1016/j.joca.2013.08.011 PMC3815567 23954774

[6] van Weeren PR , Barneveld A . The effect of exercise on the distribution and manifestation of osteochondrotic lesions in the warmblood foal. Equine Vet J Suppl. 1999;31:16‐25. 10.1111/j.2042-3306.1999.tb05309.x 10999656

[7] McCoy AM , Ralston SL , McCue ME . Short‐ and long‐term racing performance of Standardbred pacers and trotters after early surgical intervention for tarsal osteochondrosis. Equine Vet J. 2014;47:438‐444. 24819047 10.1111/evj.12297 PMC4229490

[8] Boorman S , Hofmeister EH , Ross MW , et al. Influence of osteochondrosis on the longevity and racing performance of standardbred trotters and pacers. Vet Surg. 2021;50:507‐516. 33460472 10.1111/vsu.13568

[9] R Core Team . R: A Language and Environment for Statistical Computing. R Foundation for Statistical computing; 2024 https://www.R-project.org/

[10] Therneau TM , Grambsch PM . Modeling Survival Data: Extending the Cox Model. Springer; 2000.

[11] Therneau T . A package for survival analysis in R. R package version 3.7‐0. 2024 https://CRAN.R-project.org/package=survival

[12] Kassambara A , Kosinki M , Biecek P . Survminer: Drawing Survival Curves Using “ggplot2”. R package version 0.4.9. 2021 https://CRAN.R-project.org/package=survminer

[13] Lykkjen S , Roed KH , Dolvik NI . Osteochondrosis and osteochondral fragments in Standardbred trotters: prevalence and relationships. Equine Vet J. 2012;44:332‐338. 21895752 10.1111/j.2042-3306.2011.00434.x

[14] Dik KJ , Enzerink E , van Weeren PR . Radiographic development of osteochondral abnormalities, in the hock and stifle of Dutch warmblood foals, from age 1 to 11 months. Equine Vet J Suppl. 1999;31:9‐15. 10.1111/j.2042-3306.1999.tb05308.x 10999655

[15] Van Cauter R , Serteyn D , Lejeune J‐P , Rousset A , Caudron I . Evaluation of the appearance of osteochondrosis lesions by two radiographic examinations in sport horses aged from 12 to 36 months. PLoS One. 2023;18:e0286213. 37220101 10.1371/journal.pone.0286213 PMC10204974

[16] Naccache F , Metzger J , Distl O . Genetic risk factors for osteochondrosis in various horse breeds. Equine Vet J. 2018;50:556‐563. 29498750 10.1111/evj.12824

