# Supplementary material for: Natural progression of tarsal osteochondrosis in Standardbred pacers and trotters
Source: Vet Surg. 2026 Jan 3;55(4):685–92. doi: 10.1111/vsu.70073 (PMC13150056; doi:10.1111/vsu.70073)
Supplement: Supplementary file 1 — Supplementary TABLE S1: Distribution of foals between farms across the years of study enrollment. Supplemental TABLE S 2: Survival analysis statistics for incidence and healing of osteochondrosis lesions in 103 Standardbred foals, by sex, from 2 to 12 months of age. Supplemental TABLE S 3: Survival analysis statistics for incidence and healing of osteochondrosis lesions in 103 Standardbred foals, by farm, from 2 to 12 months of age. Supplemental TABLE S 4: Survival analysis statistics for incidence and healing of osteochondrosis lesions in 103 Standardbred foals, by foaling year, from 2 to 12 months of age. Supplemental TABLE S 5: Summary of observed activity by observation week. Percentages shown for each activity are the concatenation of all horses, farms, and years and are presented as median [interquartile range]. Foals were enrolled in the study at 8 weeks of age, and within each week's observations (after Week 1) there was a range in the ages of the observed foals because foaling at each farm was spread out over several weeks. Supplemental TABLE S 6: Summary of activity in percentage of observed time by farm and time period (pre‐weaning, weaning, post‐weaning). [file VSU-55-685-s001.docx]

**Supplemental Table 1:** Distribution of foals between farms across the years of study enrollment.

| **Foaling Year** | **Farm 1** | **Farm 2** | **Farm 3** | **Total (Year)** |
| --- | --- | --- | --- | --- |
| **2015** | 0 | 12 | 0 | 12 |
| **2016** | 13 | 7 | 0 | 20 |
| **2017** | 36 | 8 | 29 | 73 |
| **2018** | 0 | 14 | 29 | 43 |
| **Total (Farm)** | 49 | 41 | 58 | 148 |

**Supplemental Table 2:** Survival analysis statistics for healing of osteochondrosis lesions in 103 affected Standardbred foals, by sex, from 2-12 months of age. For this analysis, “survival” was defined as lesion persistence. Exam 0 is at the time of enrollment. Foals that were not radiographed at 4 months of age were conservatively assumed not to have healed their lesions between 2 and 4 months of age. P-value for comparison between groups was 0.19. 95% CI = 95% confidence interval.

| **Exam number** | **Age (months)** | **Horses at risk** | **Horses healed in interval** | **Proportion affected** | **Standard error** | **95% CI** |
| --- | --- | --- | --- | --- | --- | --- |
| **Colts** | | | | | | |
| 0 | 2 | 59 | 13 | 0.78 | 0.05 | 0.68, 0.89 |
| 1 | 4 | 46 | 16 | 0.51 | 0.07 | 0.40, 0.65 |
| 2 | 6 | 30 | 4 | 0.44 | 0.06 | 0.33, 0.59 |
| 3 | 8 | 26 | 3 | 0.39 | 0.06 | 0.28, 0.54 |
| 4 | 10 | 23 | 2 | 0.36 | 0.06 | 0.25, 0.50 |
| 5 | 12 | 21 | 0 | 0.36 | 0.06 | 0.25, 0.50 |
| **Fillies** | | | | | | |
| 0 | 2 | 44 | 12 | 0.73 | 0.07 | 0.61, 0.87 |
| 1 | 4 | 32 | 19 | 0.32 | 0.07 | 0.21, 0.49 |
| 2 | 6 | 13 | 0 | 0.30 | 0.07 | 0.19, 0.47 |
| 3 | 8 | 13 | 2 | 0.25 | 0.07 | 0.15, 0.42 |
| 4 | 10 | 11 | 0 | 0.25 | 0.07 | 0.15, 0.42 |
| 5 | 12 | 11 | 0 | 0.25 | 0.07 | 0.15, 0.42 |

**Supplemental Table 3:** Survival analysis statistics for healing of osteochondrosis lesions in 103 affected Standardbred foals, by farm, from 2-12 months of age. For this analysis, “survival” was defined as lesion persistence. Exam 0 is at the time of enrollment. Horses at Farm 3 were not radiographically examined at 4 months of age and were conservatively assumed not to have healed their lesions between 2 and 4 months of age. P-value for comparison between groups was 0.06. 95% CI = 95% confidence interval.

| **Exam number** | **Time (months)** | **Horses at risk** | **Horses healed in interval** | **Proportion affected** | **Standard error** | **95% CI** |
| --- | --- | --- | --- | --- | --- | --- |
| **Farm 1** | | | | | | |
| 0 | 2 | 35 | 14 | 0.60 | 0.08 | 0.46, 0.79 |
| 1 | 4 | 21 | 5 | 0.46 | 0.08 | 0.32, 0.66 |
| 2 | 6 | 16 | 1 | 0.43 | 0.08 | 0.29, 0.63 |
| 3 | 8 | 15 | 1 | 0.40 | 0.08 | 0.27, 0.60 |
| 4 | 10 | 14 | 0 | 0.40 | 0.08 | 0.27, 0.60 |
| 5 | 12 | 14 | 0 | 0.40 | 0.08 | 0.27, 0.60 |
| **Farm 2** | | | | | | |
| 0 | 2 | 29 | 11 | 0.62 | 0.09 | 0.47, 0.83 |
| 1 | 4 | 18 | 9 | 0.31 | 0.09 | 0.18, 0.53 |
| 2 | 6 | 9 | 2 | 0.24 | 0.08 | 0.13, 0.46 |
| 3 | 8 | 7 | 1 | 0.21 | 0.08 | 0.10, 0.42 |
| 4 | 10 | 6 | 1 | 0.17 | 0.07 | 0.08, 0.38 |
| 5 | 12 | 5 | 0 | 0.17 | 0.07 | 0.08, 0.38 |
| **Farm 3** | | | | | | |
| 0 | 2 | 39 | 0 | 1.00 | 0.00 | 1.00, 1.00 |
| 1 | 4 | 39 | 20 | 0.49 | 0.08 | 0.35, 0.67 |
| 2 | 6 | 19 | 2 | 0.44 | 0.08 | 0.31, 0.62 |
| 3 | 8 | 17 | 3 | 0.36 | 0.08 | 0.24, 0.55 |
| 4 | 10 | 14 | 1 | 0.33 | 0.08 | 0.21, 0.52 |
| 5 | 12 | 13 | 0 | 0.33 | 0.08 | 0.21, 0.52 |

**Supplemental Table 4:** Survival analysis statistics for healing of osteochondrosis lesions in 103 affected Standardbred foals, by foaling year, from 2-12 months of age. For this analysis, “survival” was defined as lesion persistence. Exam 0 is at the time of enrollment. Foals that were not radiographed at 4 months of age were conservatively assumed not to have healed their lesions between 2 and 4 months of age. P-value for comparison between groups was 0.64. 95% CI = 95% confidence interval.

| **Exam number** | **Time (months)** | **Horses at risk** | **Horses healed in interval** | **Proportion affected** | **Standard error** | **95% CI** |
| --- | --- | --- | --- | --- | --- | --- |
| **2015** | | | | | | |
| 0 | 2 | 10 | 3 | 0.70 | 0.14 | 0.47, 1.00 |
| 1 | 4 | 7 | 3 | 0.40 | 0.15 | 0.19, 0.86 |
| 2 | 6 | 4 | 1 | 0.30 | 0.14 | 0.11, 0.77 |
| 3 | 8 | 3 | 1 | 0.20 | 0.13 | 0.06, 0.69 |
| 4 | 10 | 2 | 1 | 0.10 | 0.09 | 0.02, 0.64 |
| 5 | 12 | 1 | 0 | 0.10 | 0.09 | 0.02, 0.64 |
| **2016** | | | | | | |
| 0 | 2 | 15 | 5 | 0.67 | 0.12 | 0.47, 0.95 |
| 1 | 4 | 10 | 3 | 0.47 | 0.13 | 0.27, 0.80 |
| 2 | 6 | 7 | 1 | 0.40 | 0.13 | 0.22, 0.74 |
| 3 | 8 | 6 | 0 | 0.40 | 0.13 | 0.22, 0.74 |
| 4 | 10 | 6 | 0 | 0.40 | 0.13 | 0.22, 0.74 |
| 5 | 12 | 6 | 0 | 0.40 | 0.13 | 0.22, 0.74 |
| **2017** | | | | | | |
| 0 | 2 | 55 | 13 | 0.76 | 0.06 | 0.66, 0.89 |
| 1 | 4 | 42 | 17 | 0.46 | 0.07 | 0.34, 0.61 |
| 2 | 6 | 25 | 3 | 0.40 | 0.07 | 0.29, 0.55 |
| 3 | 8 | 22 | 3 | 0.35 | 0.06 | 0.24, 0.50 |
| 4 | 10 | 19 | 1 | 0.33 | 0.06 | 0.22, 0.48 |
| 5 | 12 | 18 | 0 | 0.33 | 0.06 | 0.22, 0.48 |
| **2018** | | | | | | |
| 0 | 2 | 23 | 4 | 0.83 | 0.08 | 0.69, 1.00 |
| 1 | 4 | 19 | 11 | 0.35 | 0.10 | 0.20, 0.61 |
| 2 | 6 | 8 | 0 | 0.35 | 0.10 | 0.20, 0.61 |
| 3 | 8 | 8 | 1 | 0.30 | 0.10 | 0.16, 0.57 |
| 4 | 10 | 7 | 0 | 0.30 | 0.10 | 0.16, 0.57 |
| 5 | 12 | 7 | 0 | 0.30 | 0.10 | 0.16, 0.57 |

**Supplemental Table 5:** Summary of observed activity by observational week. Percentages shown for each activity are the concatenation of all horses, farms, and years and are presented as median [interquartile range]. Foals were enrolled in the study at 8 weeks of age, and within each week’s observations (after Week 1) there was a range in the ages of the observed foals because foaling at each farm was spread out over several weeks.

| **Week** | **Nursing** | **Eating/ Grazing** | **Walking** | **Trotting** | **Pacing** | **Cantering/ Galloping** | **Standing Quietly** | **Lying Down** |
| --- | --- | --- | --- | --- | --- | --- | --- | --- |
| **1** | 2.7 [2.5-3.4] | 39.6 [36.9-44.8] | 10.7 [7.9-12.8] | 0.6 [0.2-1.1] | 0.2 [0.1-0.3] | 0.6 [0.1-0.8] | 36.5 [33.3-45.7] | 6.4 [4.3-8.2] |
| **2** | 1.1 [0.5-4.8] | 37.2 [33.6-41.1] | 7.6 [7.5-12.3] | 0.8 [0.1-1.2] | 0.3 [0.1-0.7] | 0.8 [0.1-1.0] | 35.0 [33.9-46.7] | 0.6 [0.2-11.7] |
| **3** | 2.9 [0.1-4.0] | 53.4 [47.4-78.7] | 9.3 [5.5-17.2] | 0.8 [0.3-1.0] | 0.4 [0.2-1.4] | 0.4 [0.2-0.5] | 21.9 [5.9-33.2] | 0.0 [0.0-0.8] |
| **4** | 1.4 [0.8-3.6] | 49.9 [43.0-60.4] | 10.3 [9.2-11.5] | 0.4 [0.3-0.9] | 0.3 [0.1-0.5] | 0.5 [0.3-0.8] | 31.7 [22.8-39.6] | 3.6 [1.3-4.9] |
| **5** | 0.8 [0.4-1.7] | 63.2 [59.7-68.8] | 11.2 [7.8-13.4] | 0.4 [0.2-0.7] | 0.2 [0.02-0.3] | 0.1 [0.03-0.5] | 17.3 [16.0-18.8] | 2.9 [0.8-9.6] |
| **6** | 1.3 [0.7-2.1] | 46.4 [39.4-67.5] | 7.7 [7.4-10.9] | 0.2 [0.1-0.4] | 0.1 [0.0-0.3] | 0.5 [0.2-0.8] | 38.2 [18.5-43.8] | 3.6 [1.6-5.7] |
| **7** | 1.1 [0.7-1.4] | 48.9 [42.9-60.3] | 9.7 [8.6-12.5] | 0.4 [0.2-0.4] | 0.1 [0.0-0.2] | 0.3 [0.2-0.3] | 26.7 [23.2-41.5] | 3.7 [2.3-4.8] |
| **8** | 1.7 [1.0-2.1] | 59.9 [47.2-67.2] | 10.6 [4.4-14.8] | 0.3 [0.1-0.4] | 0.1 [0.02-0.1] | 0.03 [0.01-0.2] | 24.3 [14.9-31.8] | 1.5 [1.2-4.5] |
| **9** | 1.1 [0.5-1.3] | 50.8 [43.3-61.1] | 10.3 [6.2-11.6] | 0.1 [0.1-0.4] | 0.1 [0.02-0.1] | 0.2 [0.1-0.3] | 32.9 [19.3-42.4] | 4.4 [1.5-12.8] |
| **10** | 1.1 [0.2-1.6] | 48.7 [47.6-64.0] | 8.5 [6.3-10.7] | 0.3 [0.2-0.9] | 0.1 [0.04-0.1] | 0.3 [0.1-0.7] | 28.6 [16.9-35.3] | 3.9 [0.9-5.2] |
| **11** | 0.9 [0.01-1.5] | 54.2 [45.8-62.4] | 7.2 [5.9-10.0] | 0.1 [0.04-0.4] | 0.03 [0.01-0.03] | 0.1 [0.0-0.2] | 27.5 [19.8-36.3] | 3.9 [0.7-4.7] |
| **12** | 0.1 [0.1-1.5] | 58.4 [46.0-65.6] | 11.5 [9.1-12.6] | 0.1 [0.04-0.7] | 0.03 [0.01-0.1] | 0.03 [0.01-0.2] | 29.4 [17.3-34.8] | 0.4 [0.2-0.9] |
| **13** | 0.1 [0.01-0.9] | 58.9 [39.9-69.9] | 12.9 [6.8-17.5] | 0.2 [0.1-0.6] | 0.03 [0.0-0.1] | 0.5 [0.1-0.7] | 28.4 [22.1-33.3] | 0.6 [0.2-2.9] |
| **14** | 0.0 [0.0-0.01] | 46.6 [31.9-64.1] | 10.7 [9.8-20.8] | 0.2 [0.0-0.4] | 0.0 [0.0-0.03] | 0.1 [0.02-0.1] | 40.4 [16.8-54.2] | 0.0 [0.0-2.5] |
| **15** | 0.0 [0.0-0.0] | 63.9 [29.8-72.2] | 10.2 [6.9-12.6] | 0.0 [0.0-0.3] | 0.0 [0.0-0.0] | 0.0 [0.0-0.02] | 25.0 [8.5-49.8] | 0.3 [0.0-0.5] |
| **16** | 0.0 [0.0-0.0] | 43.3 [32.0-56.6] | 5.1 [2.1-11.6] | 0.02 [0.0-0.5] | 0.0 [0.0-0.02] | 0.0 [0.0-0.3] | 40.0 [29.9-62.7] | 0.0 [0.0-2.5] |
| **17** | 0.0 [0.0-0.0] | 45.5 [26.6-57.3] | 5.4 [2.6-8.7] | 0.1 [0.0-0.4] | 0.0 [0.0-0.0] | 0.0 [0.0-0.1] | 37.5 [21.4-70.1] | 0.0 [0.0-0.01] |
| **18** | 0.0 [0.0-0.0] | 45.7 [33.5-50.4] | 7.0 [2.9-13.6] | 0.2 [0.1-0.5] | 0.0 [0.0-0.0] | 0.2 [0.02-1.0] | 41.9 [34.3-60.6] | 0.0 [0.0-2.7] |
| **19** | 0.0 [0.0-0.0] | 34.6 [25.0-59.4] | 7.9 [2.7-9.8] | 0.02 [0.0-0.3] | 0.0 [0.0-0.0] | 0.04 [0.02-2.0] | 54.1 [59.8-75.0] | 0.0 [0.0-0.3] |
| **20** | 0.0 [0.0-0.0] | 49.0 [31.5-58.4] | 7.0 [2.6-8.7] | 0.1 [0.02-0.4] | 0.0 [0.0-0.0] | 0.1 [0.0-0.5] | 40.5 [28.6-64.9] | 0.0 [0.0-1.2] |
| **21** | 0.0 [0.0-0.0] | 48.0 [27.3-55.0] | 9.1 [3.6-13.3] | 0.5 [0.2-0.7] | 0.1 [0.0-0.3] | 0.7 [0.2-2.0] | 40.3 [28.4-58.0] | 0.4 [0.0-1.4] |
| **22** | 0.0 [0.0-0.0] | 27.0 [25.2-45.0] | 10.8 [7.4-12.3] | 0.3 [0.002-0.7] | 0.0 [0.0-0.04] | 0.4 [0.1-0.7] | 49.9 [27.6-64.4] | 6.8 [1.2-11.3] |
| **23** | 0.0 [0.0-0.0] | 51.0 [46.2-53.2] | 8.4 [5.7-9.1] | 0.3 [0.1-0.6] | 0.0 [0.0-0.04] | 0.5 [0.4-0.8] | 36.6 [34.3-41.8] | 2.1 [1.3-2.3] |
| **24** | 0.0 [0.0-0.0] | 32.4 [19.7-39.5] | 11.3 [9.9-11.9] | 0.9 [0.3-1.4] | 0.02 [0.0-0.04] | 0.3 [0.1-0.8] | 49.1[42.0-59.1] | 0.1 [0.04-9.2] |
| **25** | 0.0 [0.0-0.0] | 47.7 [37.1-52.2] | 9.2 [6.3-12.2] | 0.3 [0.2-0.5] | 0.0 [0.0-0.03] | 0.2 [0.1-0.4] | 41.1 [29.3-56.3] | 1.3 [0.7-4.5] |
| **26** | 0.0 [0.0-0.0] | 36.1 [18.7-42.4] | 6.7 [5.3-8.0] | 0.9 [0.2-1.4] | 0.0 [0.0-0.0] | 1.2 [0.2-3.6] | 46.6 [40.0-64.9] | 2.3 [0.0-4.8] |
| **27** | 0.0 [0.0-0.0] | 26.5 [21.7-36.1] | 7.7 [7.0-12.2] | 0.2 [0.1-1.0] | 0.0 [0.0-0.01] | 0.7 [0.1-1.1] | 49.1 [46.2-71.0] | 1.1 [0.2-3.2] |
| **28** | 0.0 [0.0-0.0] | 33.0 [29.3-33.2] | 7.4 [5.9-12.9] | 0.2 [0.1-0.4] | 0.0 [0.0-0.1] | 0.7 [0.6-0.7] | 53.8 [41.9-57.9] | 1.5 [0.8-5.8] |
| **29** | 0.0 [0.0-0.0] | 36.9 [31.1-45.0] | 11.3 [9.1-17.1] | 1.1 [0.5-1.7] | 0.04 [0.0-0.1] | 1.5 [1.1-1.7] | 43.3 [32.9-47.5] | 10.5 [6.1-14.0] |
| **30** | 0.0 [0.0-0.0] | 28.5 [26.1-46.4] | 12.0 [9.2-12.4] | 0.1 [0.1-0.9] | 0.04 [0.02-0.2] | 1.6 [0.8-3.4] | 43.3 [32.9-47.5] | 0.9 [0.4-5.1] |
| **31** | 0.0 [0.0-0.0] | 36.2 [32.5-40.1] | 10.0 [8.9-10.4] | 0.2 [0.2-0.3] | 0.01 [0.01-0.2] | 1.1 [0.6-1.5] | 40.3 [33.4-51.2] | 2.1 [1.5-13.7] |
| **32** | 0.0 [0.0-0.0] | 49.7 [25.7-72.4] | 9.6 [8.1-11.3] | 0.8 [0.4-1.3] | 0.01 [0.0-0.6] | 2.0 [0.7-3.7] | 30.7 [14.2-51.5] | 0.1 [0.1-2.8] |
| **33** | 0.0 [0.0-0.0] | 32.1 [25.5-40.1] | 6.9 [5.7-7.9] | 0.2 [0.04-0.6] | 0.0 [0.0-0.01] | 0.2 [0.1-0.4] | 45.4 [34.9-60.0] | 1.2 [0.8-9.9] |
| **34** | 0.0 [0.0-0.0] | 20.2 [14.4-31.5] | 3.8 [3.7-8.2] | 0.4 [0.1-0.7] | 0.0 [0.0-0.3] | 0.6 [0.4-0.7] | 37.0 [36.8-41.4] | 1.3 [0.3-37.8] |
| **35** | 0.0 [0.0-0.0] | 31.7 [24.5-40.2] | 12.3 [9.6-12.9] | 1.7 [0.5-2.1] | 0.02 [0.0-0.1] | 0.6 [0.1-1.9] | 52.4 [42.2-59.2] | 2.1 [1.0-3.0] |
| **36** | 0.0 [0.0-0.0] | 25.5 [19.1-29.3] | 9.2 [6.5-13.9] | 0.5 [0.4-0.6] | 0.0 [0.0-0.1] | 0.4 [0.3-0.4] | 54.6 [51.5-58.3] | 11.1 [2.4-13.0] |
| **37** | 0.0 [0.0-0.0] | 21.2 [11.4-38.4] | 7.6 [6.9-9.9] | 0.1 [0.01-0.8] | 0.1 [0.01-0.1] | 0.3 [0.1-0.5] | 50.9 [52.3-62.8] | 1.3 [0.5-9.8] |
| **38** | 0.0 [0.0-0.0] | 27.7 [18.5-38.7] | 7.6 [3.3-11.0] | 0.1 [0.1-0.2] | 0.0 [0.0-0.0] | 0.9 [0.1-1.1] | 60.9 [52.3-62.8] | 0.3 [0.0-17.0] |
| **39** | 0.0 [0.0-0.0] | 23.1 [13.5-24.9] | 5.9 [5.3-9.4] | 0.2 [0.1-0.5] | 0.0 [0.0-0.1] | 0.7 [0.1-1.8] | 51.1 [50.1-58.1] | 14.7 [7.3-24.6] |
| **40** | 0.0 [0.0-0.0] | 26.1 [17.5-33.9] | 11.3 [6.3-15.6] | 0.6 [0.3-0.9] | 0.03 [0.0-0.2] | 0.7 [0.3-1.3] | 54.4 [42.8-72.2] | 3.9 [2.0-6.8] |
| **41** | 0.0 [0.0-0.0] | 22.1 [9.0-35.5] | 6.5 [5.5-8.5] | 0.5 [0.2-1.8] | 0.1 [0.0-0.2] | 1.2 [0.4-3.3] | 63.8 [47.1-74.2] | 2.0 [0.1-6.0] |
| **42** | 0.0 [0.0-0.0] | 12.5 [6.7-25.0] | 10.9 [5.6-14.5] | 0.8 [0.2-3.0] | 0.0 [0.0-0.03] | 0.7 [0.2-0.9] | 64.4 [44.5-79.4] | 4.9 [1.6-23.3] |
| **43** | 0.0 [0.0-0.0] | 6.8 [2.0-14.4] | 8.6 [5.6-9.8] | 0.2 [0.01-0.6] | 0.0 [0.0-0.004] | 0.1 [0.0-0.4] | 77.2 [69.1-88.2] | 3.5 [2.7-3.9] |
| **44** | 0.0 [0.0-0.0] | 32.5 [18.3-37.7] | 11.3 [8.9-14.6] | 0.5 [0.2-0.7] | 0.0 [0.0-0.0] | 0.5 [0.1-1.0] | 43.0 [40.9-66.9] | 1.0 [0.1-7.3] |
| **45** | 0.0 [0.0-0.0] | 47.6 [31.9-59.0] | 10.9 [10.6-13.9] | 0.5 [0.2-0.7] | 0.0 [0.0-0.1] | 0.6 [0.2-0.7] | 45.0 [28.3-48.9] | 0.0 [0.0-3.3] |
| **46** | 0.0 [0.0-0.0] | 9.5 [2.9-39.5] | 11.5 [9.6-15.4] | 0.6 [0.4-1.4] | 0.0 [0.0-0.0] | 0.9 [0.4-2.2] | 77.1 [44.6-82.9] | 0.6 [0.0-0.9] |
| **47** | 0.0 [0.0-0.0] | 29.0 [19.4-39.3] | 7.9 [5.6-10.4] | 0.2 [0.03-0.3] | 0.0 [0.0-0.0] | 0.3 [0.02-1.7] | 55.5 [47.6-64.6] | 1.1 [0.2-4.7] |
| **48** | 0.0 [0.0-0.0] | 29.6 [26.8-38.8] | 12.5 [10.4-14.7] | 0.2 [0.1-0.3] | 0.0 [0.0-0.0] | 0.4 [0.2-1.4] | 50.8 [37.7-56.5] | 1.3 [0.8-6.1] |
| **49** | 0.0 [0.0-0.0] | 36.6 [18.7-51.8] | 11.7 [8.6-13.6] | 0.4 [0.3-0.8] | 0.02 [0.004-0.1] | 0.2 [0.03-0.2] | 37.0 [27.1-55.1] | 2.2 [0.2-6.8] |

**Supplementary Table 6:** Summary of activity in percentage of observed time by farm and time period (pre-weaning, weaning, post-weaning). Values are reported as median (range). P-value is based on 2-way analysis of variance; values in **bold** are significant. Superscript symbols reflect pairwise comparisons by Tukey HSD. Horses at Farm 1 were stalled during weaning and have no data for this period.

| **Activity** | **Farm 1** | **Farm 2** | **Farm 3** | **Combined** | **p-value**  **(farm)** | **p-value (time)** |
| --- | --- | --- | --- | --- | --- | --- |
| *Pre-weaning* | | | | | |  |
| Nursing | 0.49  (0.29 – 0.69) | 1.8  (0.67 – 3.4) | 1.9  (1.1 – 2.7) | 1.1^‡^  (0.29 – 3.4) | 0.59 | **0.001** |
| Eating/  Grazing | 57.7  (54.6 – 60.8) | 53.4  (49.1 – 60.9) | 48.5  (38.0 – 59.1) | 54.5^‡^  (38.0 – 60.9) | 0.18 | **0.0007** |
| Walking | 13.8  (9.9 – 17.6) | 7.9  (7.0 – 9.8) | 11.9  (10.8 – 13.0) | 9.9  (6.8 – 17.6) | 0.39 | 0.81 |
| Trotting/  Pacing | 0.73  (0.72 – 0.74) | 0.47  (0.40 – 0.82) | 0.86  (0.66 – 1.0) | 0.72  (0.40 – 1.05) | 0.64 | 0.48 |
| Cantering/  Galloping | 0.22  (0.20 – 0.25) | 0.35  (0.31 – 0.47) | 0.73  (0.50 – 0.98) | 0.35  (0.20 – 0.98) | 0.27 | 0.07 |
| Standing Quietly | 25.8  (19.6 – 32.0) | 29.3  (23.2 – 34.8) | 29.7  (20.8 – 38.7) | 29.3^‡^  (19.6 – 38.7) | 0.07 | **0.001** |
| Lying Down | 1.2  (0.57 – 1.8) | 5.7  (5.2 – 5.9) | 6.3  (4.4 – 8.2) | 5.2  (0.57 – 8.2) | 0.29 | 0.40 |
| *Weaning* | | | | | | |
| Nursing | N/A | 0  (0 – 0) | 0.05  (0 – 0.1) | 0^†^  (0 – 0.1) |  | |
| Eating/  Grazing | N/A | 45.2  (43.7 – 52.7) | 53.1  (49.9 – 56.3) | 49.9^‡^  (43.7 – 56.3) |  |  |
| Walking | N/A | 7.7  (6.0 – 14.1) | 12.0  (10.4 – 13.6) | 10.4  (6.0 – 14.1) |  |  |
| Trotting/  Pacing | N/A | 0.66  (0.58 – 0.75) | 0.60  (0.26 – 0.95) | 0.66  (0.26 – 0.95) |  |  |
| Cantering/  Galloping | N/A | 1.1  (0.37 – 1.4) | 0.88  (0.23 – 1.5) | 1.1  (0.23 – 1.5) |  |  |
| Standing Quietly | N/A | 39.0  (31.9 – 44.4) | 28.5  (25.7 – 31.3) | 31.9^‡,†^  (25.7 – 44.4) |  |  |
| Lying Down | N/A | 1.3  (0.69 – 8.5) | 4.9  (2.6 – 7.1) | 2.6  (0.69 – 8.5) |  |  |
| *Post-weaning* | | | | | | |
| Nursing | 0  (0 – 0) | 0  (0 – 0) | 0  (0 – 0) | 0^†^  (0 – 0) |  | |
| Eating/  Grazing | 15.0  (7.8 – 22.3) | 33.2  (28.8 – 34.4) | 41.3  (40.4 – 42.3) | 33.2^†^  (7.8 – 42.3) |  |  |
| Walking | 7.3  (6.6 – 7.9) | 11.3  (7.4 – 12.8) | 11.2  (10.8 – 11.5) | 10.8  (6.6 – 12.8) |  |  |
| Trotting/  Pacing | 0.49  (0.35 – 0.62) | 0.98  (0.72 – 1.1) | 0.81  (0.73 – 0.89) | 0.73  (0.35 – 1.1) |  |  |
| Cantering/  Galloping | 1.0  (0.89 – 1.2) | 0.65  (0.53 – 0.83) | 1.4  (1.0 – 1.8) | 0.89  (0.53 – 1.8) |  |  |
| Standing Quietly | 70.9  (62.7 – 79.2) | 50.7  (45.0 – 52.1) | 40.1  (34.7 – 45.5) | 50.7^†^  (34.7 – 79.2) |  |  |
| Lying Down | 4.2  (2.9 – 5.5) | 6.2  (5.8 – 6.2) | 5.6  (0.70 – 10.5) | 5.8  (0.70 – 10.5) |  |  |
